# Outcomes and predictors of relapse and severe pneumonia in Chinese patients with AQP4-IgG-positive neuromyelitis optica spectrum disorder receiving inebilizumab: a prospective cohort study

**DOI:** 10.3389/fimmu.2025.1718896

**Published:** 2026-01-12

**Authors:** Wenjuan Huang, Hongmei Tan, Jingzi ZhangBao, Liang Wang, Yuxin Fan, Zhouzhou Wang, Chongbo Zhao, Jiahong Lu, Qiang Dong, Lei Zhou, Chao Quan

**Affiliations:** 1 Department of Neurology, Huashan Hospital, Shanghai Medical College, Fudan University, Shanghai, China; 2 National Center for Neurological Disorders (NCND), Shanghai, China; 3 Rare Disease Center, Huashan Hospital, Shanghai Medical College, Fudan University, Shanghai, China

**Keywords:** neuromyelitis optica spectrum disorder, AQP4-IgG, inebilizumab, efficacy, safety, early relapse, pneumonia

## Abstract

**Background:**

Inebilizumab was included in China’s national reimbursement drug list in 2023, enhancing its accessibility. However, there is a paucity of real-world data evaluating its performance in Chinese patients with aquaporin-4 immunoglobulin G–seropositive neuromyelitis optica spectrum disorder (AQP4-IgG+ NMOSD). This prospective study therefore sought to assess the treatment outcomes and identify predictive factors for relapse and severe pneumonia in Chinese patients treated with Inebilizumab.

**Methods:**

This study enrolled AQP4-IgG+ NMOSD patients receiving ≥1 dose of inebilizumab from March 2023 to June 2025. The primary outcome was time to first relapse. Secondary outcomes included annualized relapse rate (ARR), Expanded Disability Status Scale (EDSS) score, drug retention rates, and adverse events (AEs). Multivariate regression analyses were conducted to identify predictors of relapse and severe pneumonia.

**Results:**

Among 136 patients (91.9% female; mean age 40.3 years; median treatment duration 13.8 months), the 12-month relapse-free rate was 88.1% (95% confidence interval [CI] =82.1–94.5%). Thirteen patients experienced 14 relapses, with 7 occurring within the first 6 months. The median time to first relapse was 5.2 months (range, 0.3–11.8). Risk factors for relapse within 12 months included: age at onset ≤ 20 years (hazard ratio [HR] 8.17, 95% CI = 1.96–34.03, p=0.004), disease duration >5 years (HR 8.06, 95% CI = 1.72–37.83, p=0.008), and pre-treatment relapses (HR 3.04, 95% CI = 1.32–6.98, p=0.009). The annualized relapse rate (ARR) significantly decreased (1.496 vs 0.117, p<0.001). The median EDSS score decreased (from 2.5 to 2.0, p = 0.002) in patients who started inebilizumab during the remission phase. The 12-month drug retention rate was 88.9% (95% CI 82.8–95.5%). Seven patients developed severe pneumonia, significantly associated with baseline EDSS ≥6.5 (odds ratio [OR] 25.4, 95% CI = 2.12–334, p=0.013) and IgG <6 g/L (OR 14.2, 95% CI = 1.79–136, p=0.007).

**Conclusion:**

This study confirms the real-world effectiveness of inebilizumab in Chinese NMOSD patients but identifies distinct clinical profiles for relapse and infection risk. Patients with early-onset, long-standing, or highly active disease are at greater risk of breakthrough relapses, whereas those with high disability and hypogammaglobulinemia require vigilance for severe pneumonia. These findings advocate for a risk-stratified management approach to optimize treatment outcomes.

## Introduction

Neuromyelitis optica spectrum disorder (NMOSD) is a rare autoimmune disease characterized by inflammatory attacks on the optic nerves, spinal cord, and brainstem (1, 2). Most patients suffer relapses leading to cumulative neurological disability (3, 4). Approximately 60-80% of cases are associated with anti-aquaporin-4 (AQP4) antibodies (5–7), which induce complement-mediated astrocyte damage, demyelination, and neuronal injury (2, 8, 9). In addition to antibody production, B cells contribute to pathogenesis via cytokine secretion and T-cell activation (10).

Targeted B-cell depletion therapies, complement inhibitors, and interleukin-6 (IL-6) receptor antagonists, have markedly advanced the treatment landscape for NMOSD (11–15). Inebilizumab, a humanized anti-CD19 monoclonal antibody, demonstrated efficacy in reducing the risk of relapse in AQP4-IgG+NMOSD during the pivotal phase 2/3 N-MOmentum trial. The incidence of relapse was 11% (18 of 161) in the inebilizumab group, compared with 42% (22 of 52) in the placebo group (HR 0.227; 95% CI 0.121 to 0.423; p < 0.0001) (11). Results from the open-label extension showed that the majority of attacks (63%, 40/63) occurred during the first year of treatment. Annualized attack rates decreased year by year, and 77% (36/47) of patients remained relapse-free at 4 years (12). Both the phase 2/3 N-MOmentum trial and its open-label extension demonstrated that inebilizumab also provided sustained efficacy in reducing disability progression, hospitalizations, and magnetic resonance imaging (MRI) lesion activity (11, 12).

Furthermore, its safety profile has been characterized that inebilizumab shown similar types and frequencies of treatment-emergent adverse events with placebo and long-term inebilizumab treatment was generally well tolerated. Treatment-emergent adverse event was reported by 208 (92%) of 225 participants, only 46 (20%) were serious. Ten (4%) had at least one inebilizumab-related serious treatment-emergent adverse event, with infection accounts for the most common type (70%, 7/10). Treatment discontinuation due to inebilizumab-related treatment-emergent adverse events was reported in seven (3%) of 225 participants. Pneumonia was the most common reason (n=2), followed by one case each of myasthenia gravis, neutropenia, steroid-withdrawal syndrome (unrelated to NMOSD attack), hepatic steatosis, worsened liver function test, and female breast cancer (11, 12).

Inebilizumab was approved in China for the treatment of AQP4-IgG+ NMOSD in 2023. However, as the pivotal N-MOmentum trial and its extension study did not include Chinese patients, a significant gap remains in the real-world evidence for this population, despite the established efficacy and safety profile from global trials. To optimize early clinical management, the first year of treatment is critical for assessing initial drug response and tolerability. Therefore, prospective data focused specifically on this phase are indispensable to provide tailored guidance and define the initial benefit-risk profile.

Here, we conducted a prospective cohort study to (1) evaluate the real-world effectiveness of inebilizumab in preventing relapses and attenuating disability progression over 12 months (2); characterize its safety profile; and (3) identify predictors associated with early disease relapse and the occurrence of severe pneumonia during treatment.

## Methods

### Study design and participants

This prospective cohort study enrolled 136 consecutive patients with AQP4+NMOSD who initiated inebilizumab therapy at Department of Neurology, Huashan Hospital, between March 2023 and June 2025. Eligibility criteria were as follows: (1) age ≥18 years at enrollment; (2) diagnosis of AQP4-IgG+NMOSD according to the 2015 International Panel for NMO Diagnosis (IPND) criteria (1); (3) documented administration of at least one therapeutic dose (300 mg) of inebilizumab; and (4) availability of pre-treatment clinical data.

Intravenous inebilizumab was administered at a fixed dose of 300 mg on days 1 and 15, followed by 300 mg every 6 months thereafter. Demographic characteristics and disease history prior to inebilizumab treatment were collected, including age at disease onset, sex, attack type at onset, subsequent attack types, AQP4-IgG titer, concomitant autoimmune diseases, and details of previous treatments (including start and stop dates and the reason for treatment change).We also collected history of treatment failure, which was defined as the occurrence of one or more relapses during administration of a standard therapeutic regimen. The standard regimens for the most commonly used therapies were defined as follows: azathioprine (AZA) at 2–3 mg/kg/day, mycophenolate mofetil (MMF) at 20 mg/kg/day, tacrolimus (TAC) at 3 mg/day, and for rituximab (RTX), the low-dose regimen used in our center (100 mg intravenously on Day 1 followed by 500 mg on Day 2 for the initial cycle, with subsequent 500 mg maintenance doses administered at 6-month intervals).

Assessments followed a predefined protocol with scheduled visits at baseline (Day 1), Day 15, Month 6, and Month 12 (see **Supplementary Table 1**). Blood tests at all time points included complete blood count, liver and renal function, lipid profile, immunoglobulin isotyping (IgG, IgM, IgA, IgE), and CD20+ B-cell percentage in lymphocytes. Serum AQP4-IgG titer was measured at baseline and every 6 months thereafter.

At baseline, Expanded Disability Status Scale (EDSS) score, Modified Rankin Scale (mRS), and Hauser Ambulation Index (HAI) were assessed. These measures were subsequently repeated at Month 6 and Month 12. Chest computed tomography (CT) were performed at baseline, and again at both Month 6 and Month 12. Neuroaxis MRI was conducted at baseline and at Month 12. Adverse events (AEs) were documented using the Medical Dictionary for Regulatory Activities (MedDRA v27.1) during all visits. AE severity was graded using a 5-tier classification system: Grade 1 (mild), Grade 2 (moderate), Grade 3 (severe), Grade 4 (life-threatening), and Grade 5 (fatal). Serious adverse events (SAEs) were defined as meeting any of the following criteria (1): Grade 4–5 severity, (2) requiring hospitalization or prolongation of existing hospitalization, or (3) resulting in persistent or significant disability/incapacity. Among the SAEs, pneumonia that led to hospitalization, intravenous antibiotics, oxygen requirement, ICU admission or death was designated as severe pneumonia.

Our analysis was reported according to the Strengthening the Reporting of Observational Studies in Epidemiology criteria for observational studies (16). 

### Antibody testing

AQP4-IgG was detected by fixed cell-based indirect immunofluorescence test with HEK293 cells transfected with the M1 isoform of AQP4 at Kindstar Medical Diagnostic Laboratory, a leading provider of specialized medical testing services in China.

### Outcome measures

The primary outcome was time to first relapse within the 12-month observation period. Each potential relapse event was independently reviewed by two neurologists blinded to the patient’s treatment assignment and clinical course. A relapse was confirmed only upon consensus between the two adjudicators, based on the following criteria: new or worsening neurological dysfunction lasting over 24 hours, occurring ≥30 days after a previous attack; absence of fever, infection, or other alternative causes; objective neurological signs (e.g., decreased muscle strength in myelitis, or visual acuity/field deficits in optic neuritis); and, for worsening pre-existing symptoms, isolated sensory complaints, or brainstem syndromes, corresponding new or enhancing lesions on MRI. The severity of each attack was quantified using the Opticospinal Impairment Score (OSIS) and subsequently classified into major or minor attacks (15).

Secondary outcomes included: annualized relapse rate (ARR) at Month 12; EDSS score at Month 12 and baseline-to-Month 12 changes in EDSS, mRS, and HAI scores; treatment persistence; and AEs during treatment. Exploratory outcomes included longitudinal analyses of serological biomarkers (AQP4-IgG titers, CD20+ B-cell percentage, and immunoglobulins [IgG/IgA/IgM/IgE]); and cumulative active MRI lesions (new/enlarging T2 or gadolinium-enhancing lesions across the optic nerve, brain, and spinal cord).

### Statistical analysis

Continuous variables are summarized as mean(standard deviation, SD) or median (range), based on data distribution. Categorical variables are presented as frequencies and percentages (n, %). Between-group comparisons employed Chi-squared or Fisher’s exact tests for categorical variables, independent t-tests or one-way ANOVA for normally distributed continuous variables, and the Mann-Whitney U test or Kruskal-Wallis test for non-normally distributed variables. Longitudinal within-group comparisons were primarily conducted using the paired t-test (for normal data) or the Wilcoxon signed-rank test (for non-normal data). For analyses involving more than two time points, the repeated-measures ANOVA (for normal data) or the Friedman test (for non-normal data) was employed, with *post-hoc* tests (Bonferroni or Dunn’s) for multiple comparisons.

This single-arm trial enrolled a minimum of 85 patients, as determined by a sample size calculation detailed in the **Supplementary Materials**. The primary efficacy endpoint, time to first relapse by Month 12, was analyzed using the Kaplan-Meier method. The safety analysis set included all participants who received at least one dose of inebilizumab. Efficacy and exploratory analyses were performed on the full analysis set, which comprised patients with a minimum of 12 months of follow-up data.

The ARR was calculated as the number of relapses per patient-year. Bootstrapping with 1,000 replicates was used to estimate the 95% confidence interval (CI). For patients with a pretreatment disease duration of less than 12 months, the pretreatment ARR was annualized over a 12-month period. The post-treatment ARR analysis included only patients with ≥12 months of follow-up after initiating inebilizumab.

Predictors of time to relapse were identified using Cox proportional hazards regression. Variables with a univariable p-value < 0.20 were included in the multivariable model. The proportional hazards assumption was verified by examining Schoenfeld residuals (global test p > 0.05) and log-minus-log survival plots. Results are reported as hazard ratios (HRs) with 95% CIs.

AEs were summarized by incidence (%) and exposure-adjusted incidence rates (events per patient-year; 95% CI). Risk factors for severe pneumonia (a binary outcome) were assessed using logistic regression. All pre-specified baseline covariates—including age at treatment initiation, sex, body mass index (BMI), baseline EDSS score, comorbidities (e.g., diabetes, cardiovascular diseases), baseline serum IgG level, rate of serum IgG decline, history of high-dose steroid pulse therapy, baseline oral steroid dosage, and prior immunosuppressant use—were included in a multivariable model. Backward stepwise selection was employed using a threshold of p < 0.20 for variable retention. The goodness-of-fit of the final model was evaluated using the Hosmer-Lemeshow test. Results are presented as odds ratios (OR) with 95%CI. The variance inflation factor (VIF) was computed for each variable to diagnose multicollinearity in the multivariable Cox and logistic regression models. The following established thresholds were applied: a VIF < 5 was deemed indicative of acceptable collinearity, a VIF between 5 and 10 was considered to represent moderate multicollinearity, and a VIF ≥ 10 was considered indicative of severe multicollinearity that could distort the model estimates (17).

All statistical analyses were performed using GraphPad Prism version 10.1.2 and R software version 4.5.0. A two-tailed p-value < 0.05 was considered statistically significant.

## Results

### Demographic and clinical features

Baseline characteristics are summarized in **Table 1**. The study enrolled 136 participants, with a mean age of 40.3 ± 11.5 years at the time of inebilizumab initiation, of whom 91.9% were female. Following inebilizumab initiation, the median treatment duration was 13.8 months (range: 1.0–28.0 months). Among these participants, 87 patients received inebilizumab for ≥12 months. Inebilizumab was used as first-line maintenance therapy in 42.6% (58/136) of the patients. Seventy patients were treated with inebilizumab during the acute phase (<3 months post-attack), and 66 patients initiated treatment during the remission phase. Concurrent baseline prednisolone therapy was administered to 119 participants (87.5%) at a median daily dose of 30 mg (range: 0–65 mg).

**Table 1:** Baseline demographic and clinical characteristics of the study participants.

Characteristic	The whole population (n=136)
Age at inebilizumab initiation, years,mean (SD)	40.3 (11.5)
Gender, *n* (%)	
Female	125 (91.9)
Male	11 (8.1)
Disease duration, months, median (range)	32.0 (1.0-401.0)
Concomitant autoimmune diseases, *n* (%)	36 (26.4)
Type of most recent attack, *n* (%)	
Optic neuritis	56 (41.1)
Myelitis	54 (40.0)
Brain or brainstem	8 (5.8)
Mixed	18 (13.1)
Number of attacks before inebllizumab therapy, median (range)	2 (1–20)
Baseline EDSS score, median (range)	2.5 (0-8.0)
Baseline mRs score, median (range)	1 (0-5)
Baseline HAI, median (range)	1 (1-11)
Number of maintenance therapy, *n* (%)	
0	58 (42.6)
1	46 (33.8)
2	21 (15.5)
3	10 (7.4)
4	1 (0.7)
Concurrent use of prednisolone, n (%)	119 (87.5)
Baseline prednisolone dosage, mg/d, median (range)	30 (0-65)
Previous maintenance therapy^†^, *n* (%)	
Non-biological immunosuppression	50 (37.0)
Azathioprine	17 (12.6)
Mycophenolate mofetil	37 (27.4)
Tacrolimus	8 (5.9)
Cyclosporin A	4 (3.0)
Cyclophosphamide	2 (1.5)
Autologous Stem Cell Transplantation	1 (0.7)
Biological agent, *n* (%)	47 (34.8)
Rituximab	30 (22.2)
Eculizumab	12 (8.9)
Ofatumumab	4 (3.0)
Interferon-β	2 (1.5)
Tocilizumab	1 (0.7)
Previous NMOSD treatment^†^, *n* (%)	
Any therapy	109 (80.5)
High-dose methylprednisolone pulse therapy	104 (76.5)
Intravenous immunoglobulin	20 (14.7)
Plasmapheresis	13 (9.6)
Eculizumab	12 (8.9)

†Patients could receive more than one type of previous treatment

AQP4, aquaporin 4; EDSS, Expanded Disability Status Scale; mRS, Modified Rankin Scale; HAI, Hauser and Ambulation Index; NMOSD, neuromyelitis optica spectrum disorder.

### Time to first attack

**Figure 1:**
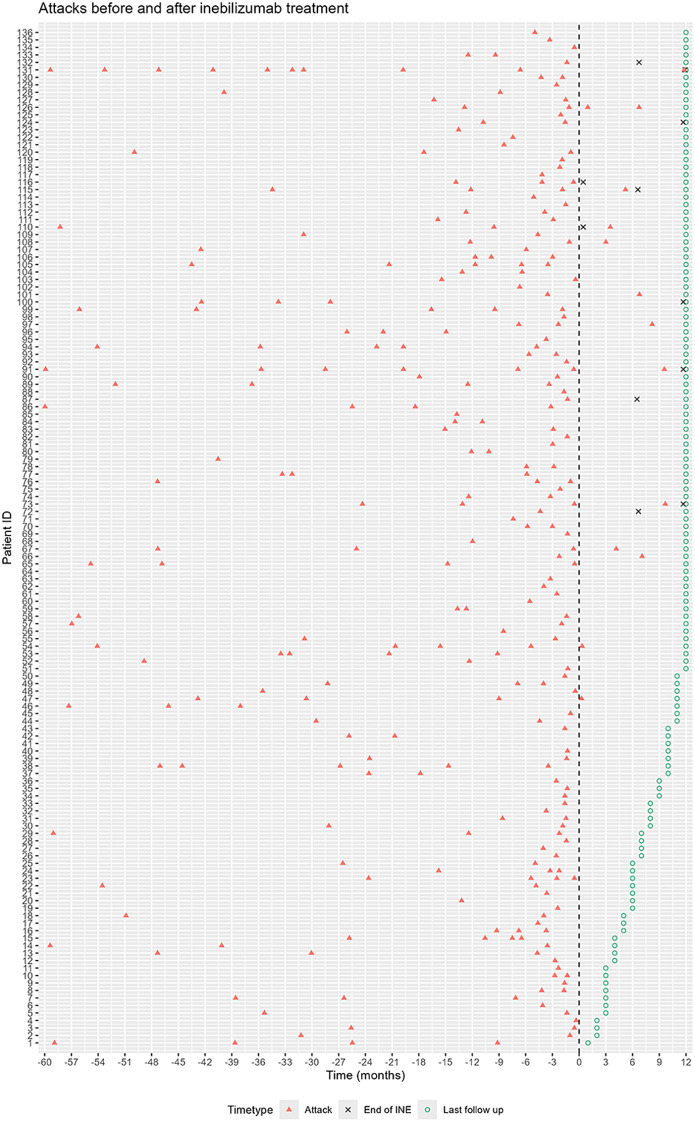
Relapse five year before and one year after initial of inebilizumab. The vertical line (Time 0) indicates treatment initiation. Each row of symbols on the y-axis represents a patient. x-axis: time relative to start of immunosuppressive treatment (months). y-axis: patients. For patients with treatment duration exceeding 12 months, only the first 12 months of data are shown. INE, inebilizumab.

**Figure 2:**
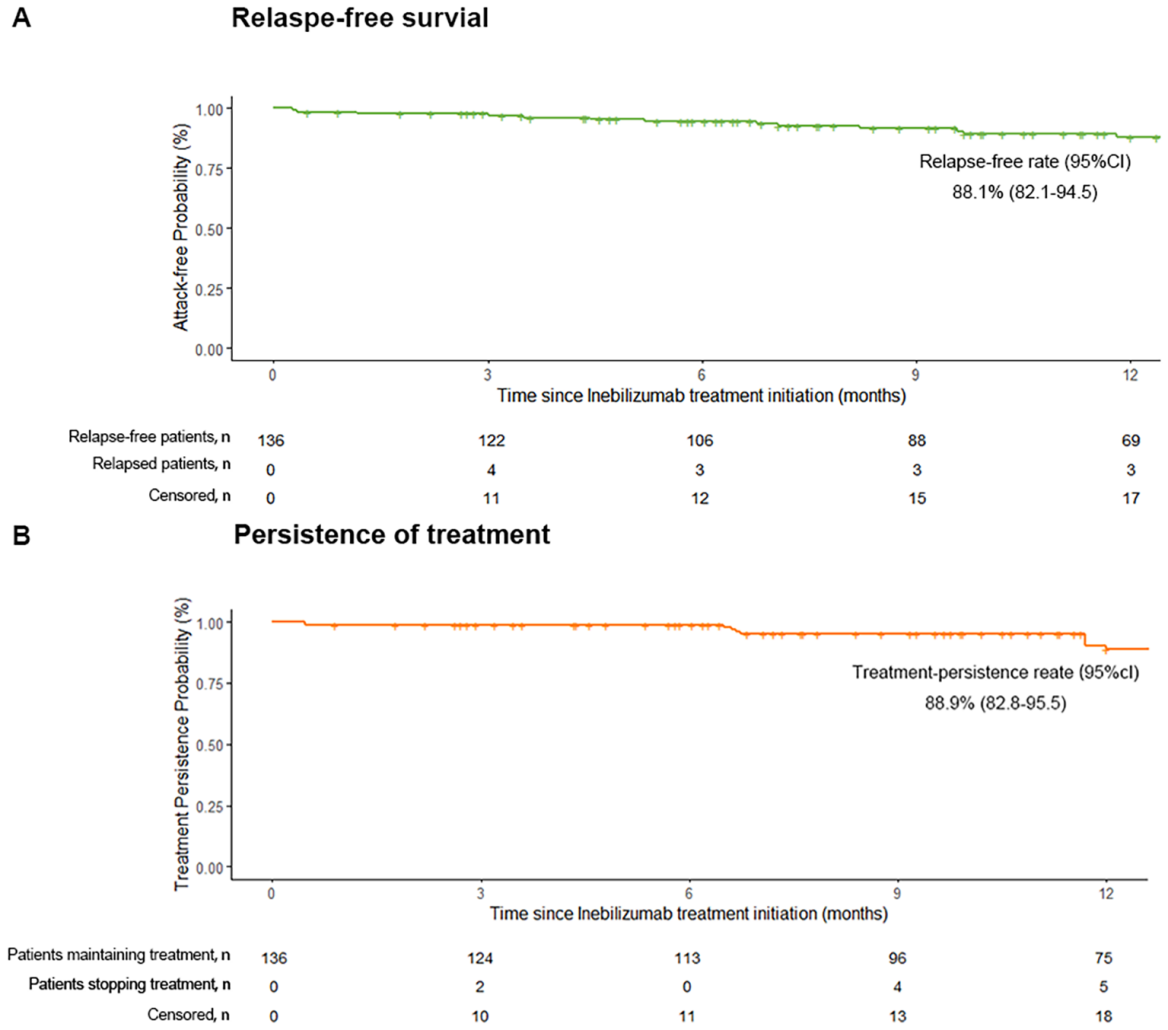
Kaplan–Meier analysis of clinical outcomes with inebilizumab therapy. **(a)** 12-Month Relapse-Free Survival with Inebilizumab, **(b)** 12-Month Treatment Persistence of Inebilizumab.

Attacks occurring five years before and 12 months after inebilizumab treatment are presented in **Figure 1**. During the 12-month study period, 14 attacks occurred in 13 patients, with a median time to first relapse of 5.2 months (range 0.3-11.8). Among these attacks, 7 occurring within the first 6 months after inebilizumab initiation. One patient experienced two relapses each at 1.2 and 6.7 months. The 12-month relapse-free rate was 88.1% (95% CI: 82.1-94.5%; **Figure 2A**). Myelitis (n=6) was the most common relapse type, followed by optic neuritis (n=5), area postrema syndrome (n=2), and dorsal medulla-involving myelitis (n=1). According to the OSIS, 3 attacks were classified as major and 11 as minor. Treatments for relapses were as follows: high-dose methylprednisolone pulse therapy (HDMT; n=9), oral prednisolone (n=2, dosage: 40mg and 20mg), HDMT plus plasmapheresis (n=1), HDMT plus intravenous immunoglobulin (n=1), and intravenous immunoglobulin alone (n=1). During attacks, seven patients underwent testing for CD20+ B-cell percentages among lymphocytes. Only two patients exhibited CD20+ B-cell percentages >1% (1.18% and 1.78%), while the remaining five maintained adequate B-cell depletion (0.01% [n=3] or 0% [n=2]).

### Factors associated with relapse during treatment

Patients who completed ≥ 6 months of follow-up were included to identify factors associated with relapse during the first year of inebilizumab treatment. We first compared the clinical data between patients with and without relapse and observed that patients experiencing relapse exhibited significantly younger age at disease onset (mean [SD] age 28.6 [12.7] vs. 36.6 [11.5] years; p = 0.015) and had longer disease duration before treatment initiation (median [range] 13.1 [12.0–276.8] vs. 101.7 [12.0–382.8] months; p = 0.001). A higher proportion of patients who relapsed had experienced treatment failure prior to inebilizumab (53.8% [7/13] vs. 25.5% [27/106]; p = 0.049) (**Table 2**).

**Table 2:** Clinical characteristics and factors associated with the risk of early relapse after inebilizumab initiation.

Clinical characteristics and factors	Baseline characteristics comparison			Univariate analysis*		Multivariate analysis*	
	Without relapse N = 106^*^	Relapse N = 13^*^	P value	HR (95% CI)	P value	HR (95% CI)	P value
Age at treatment initiation, years, mean (SD)	40.1 (10.9)	39.7 (13.8)	0.815	1.00 (0.95-1.05)	0.974	-	-
Onset age, years, mean (SD)	28.6 (12.7)	36.6 (11.5)	**0.015**	0.93 (0.87-0.99)	**0.028**	-	-
Onset age, n(%)			**<0.001**				
>20	102 (96.2)	8 (61.5)		Reference		Reference	
≤20	4 (3.8)	5 (38.5)		11.33 (3.67-34.94)	**<0.001**	8.17 (1.96-34.03)	**0.004**
Gender, n(%)			0.344				
Female	97 (91.5)	11 (84.6)		Reference			
Male	9 (8.5)	2 (15.4)		2.12 (0.47-9.65)	0.330	–	–
Treatment failure before Inelizumab^#^, n (%)			0.049				
No	79 (74.5)	6 (46.2)		Reference		Reference	
Yes	27 (25.5)	7 (53.8)		3.15 (1.06-9.38)	**0.040**	0.23 (0.04-1.37)	0.107
Rapid corticosteroid tapering^†^, n(%)			0.092				
No	103 (97.2)	11 (84.6)		Reference		Reference	
Yes	3 (2.8)	2 (15.4)		5.18 (1.14-23.44)	**0.033**	4.89 (0.86-27.82)	0.073
Onset type, n(%)			0.186				
Optic neuritis	55 (51.9)	7 (53.8)		Reference		-	-
Myelitis	28 (26.4)	2 (15.4)		0.55 (0.11-2.66)	0.459	-	-
Brainstem/cerebral	12 (11.3)	4 (30.8)		2.43 (0.71-8.31)	0.156	-	-
Mixed	11 (10.4)	0 (0)		0.00 (0.00-Inf)^&^	0.998	-	-
Recent relapse type, n(%)			0.687				
Optic neuritis	44 (41.6)	8 (61.5)		Reference			
Myelitis	42 (39.6)	4 (30.8)		0.56 (0.17-1.87)	0.348		
Brainstem/cerebral	8 (7.5)	0 (0)		0.00 (0.00-Inf)^&^	0.998		
Mixed, n(%)	12 (11.3)	1 (7.7)		0.51 (0.06-4.07)	0.525		
Number of attacks one year before inbelizumab treatment, median (range)	1 (0-3)	1 (1-2)	0.189	1.58 (0.70-3.57)	0.274	–	–
Number of attacks two year before inbelizumab treatment, median (range)	1 (0-4)	1 (1-4)	**0.008**	2.04 (1.20-3.47)	**0.008**	3.04 (1.32-6.98)	**0.009**
Time from recent attack to inbelizumab initiation, months, median (range)	3.1 (0.4-62.0)	2.3 (0.4-9.7)	0.347	0.93 (0.79-1.08)	0.333	-	-
Disease duration before inbelizumab treatment, months, median (range)	13.2 (0.5-276.8)	101.7 (2.2- 382.2)	**0.001**	1.01 (1.00-1.01)	**<0.001**	-	-
Disease duration before inbelizumab treatment ≤5 year, n(%)	91 (85.8)	5 (38.5)	**<0.001**	Reference		Reference	
Disease duration before inbelizumab treatment >5 year, n(%)	15 (14.2)	8 (61.5)	**0.043**	8.20 (2.67-25.13)	**<0.001**	8.06 (1.72-37.83)	**0.008**
Concomitant autoimmune diseases, n (%)	33 (31.1)	2 (15.3)	**<0.001**	0.41 (0.09-1.83)	0.242	-	-
EDSS at baseline, median (range)	2.5 (0.0-8.0)	3.0 (1.0-5.0)	0.319	1.10 (0.78-1.54)	0.586	–	–
EDSS at baseline, n(%)			0.474				
≤3	60 (56.6)	6 (46.2)		Reference			
>3	46 (43.4)	7 (53.8)		1.50 (0.50-4.46)	0.467		
AQP4-ab titer, n(%)			0.220				
<1000	91 (85.8)	9 (69.2)		Reference		Reference	
≥1000	15 (14.2)	4 (30.8)		2.29 (0.71-7.44)	0.168	4.32 (0.92-20.28)	0.063
Sustained CD20+B cell depletion^‡^, n(%)			0.261				
No	8 (7.5)	2 (15.4)		Reference			
Yes	98 (92.5)	11 (84.6)		0.52 (0.12-2.35)	0.396	–	–
MRI lesions at baseline, n(%)			0.290				
No	49 (46.2%)	4 (30.8%)		Reference			
Yes	57 (53.8%)	9 (69.2%)		1.86 (0.57-6.05)	0.301	–	–
Number of MRI lesions, median (range)	1 (0-12)	2.0 (0-10)	0.405	1.02 (0.90-1.17)	0.728		

^#^Treatment failure was specifically defined as the occurrence of one or more relapses during administration of a standard therapeutic regimen. In particular, for rituximab (RTX), treatment failure was assessed specifically in the context of the low-dose regimen used in our center, consisting of 100 mg intravenously on Day 1 followed by 500 mg on Day 2 for the initial cycle, with subsequent 500 mg maintenance doses administered at 6-month intervals.

**†**Defined as >5mg per week when dose >30mg or >5mg per month when dose ≤30mg

**‡**Defined as CD20+ B-cell percentage among lymphocytes <1%

&The hazard ratio is reported as 0.00 with an infinite confidence interval because no events occurred in the group.

*Data are presented as n (%), mean(sd) or median (range). Between-group comparisons were made using the χ² test, Fisher’s exact test, Student’s t-test, or the Mann–Whitney U test, as appropriate. Variables with a univariable P value < 0.20 in Cox regression analysis were included in the multivariable model.

AQP4, aquaporin 4; EDSS, Expanded Disability Status Scale; MRI, magnetic resonance imaging.Bold values indicate statistical significance (p < 0.05).

We assessed multicollinearity among the independent variables using VIF. The VIF values ranged from 1.20 to 2.62, all well below the conservative threshold of 5.0, indicating the absence of substantial multicollinearity in our multivariable Cox regression model. Both univariate and multivariate analyses identified age at onset ≤20 years (HR 8.17 [1.96–34.03], p = 0.004), disease duration >5 years (HR 8.06 [1.72–37.83], p = 0.008), and attacks within two years before inebilizumab (HR 3.04 [1.32–6.98], p = 0.009) as significant factors associated with increased risk of relapse. While treatment failure (defined as breakthrough relapses during administration of a standard therapeutic regimen) during maintenance therapy prior to inebilizumab and rapid corticosteroid tapering during inebilizumab therapy (defined as >5 mg per week when dose >30 mg or >5 mg per month when dose ≤30 mg) were associated with relapse risk in univariate analysis (**Table 2**), but these association were not significant in the multivariate analysis.

### ARR

**Figure 3:**
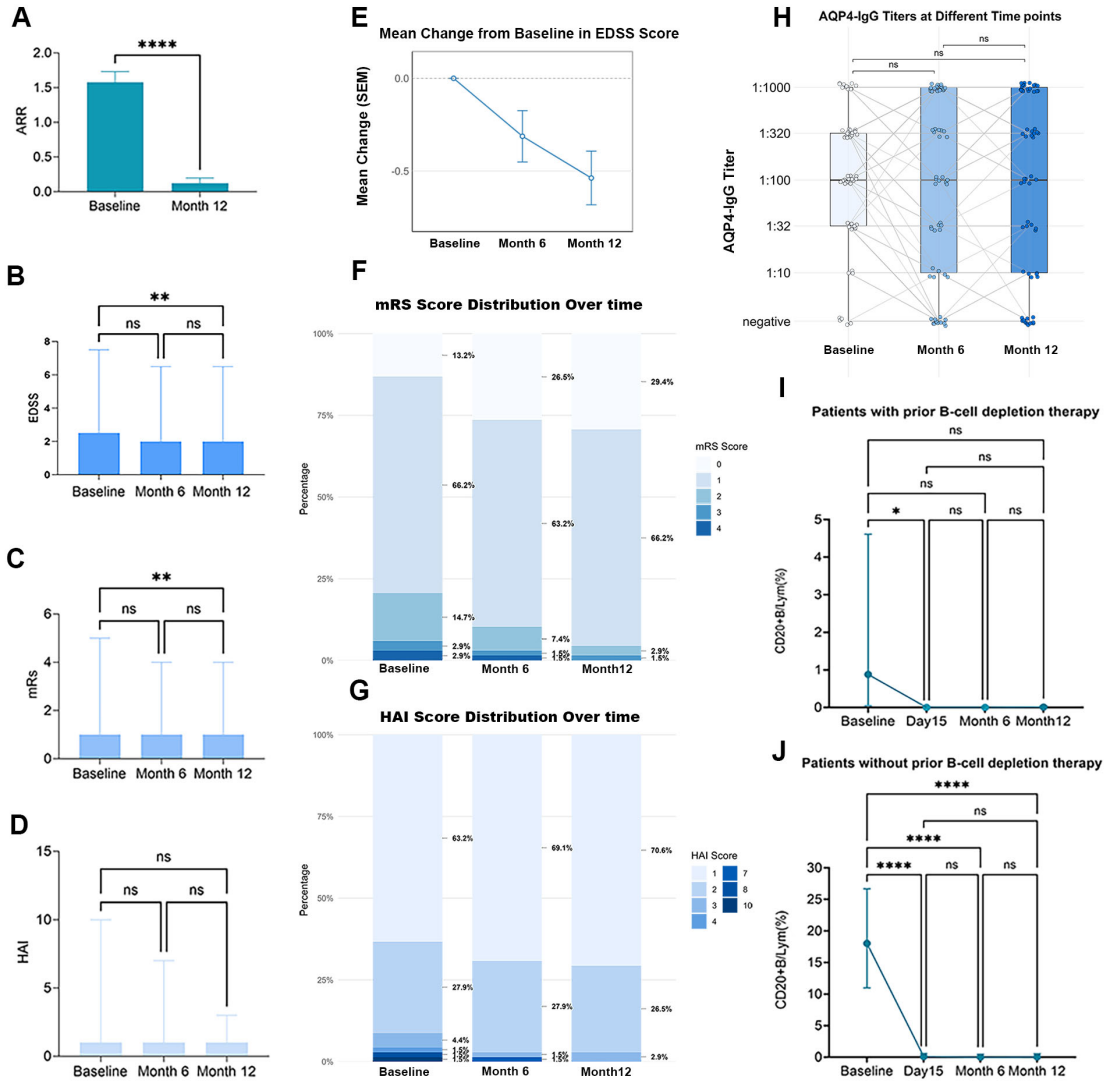
Secondary and exploratory outcome with inebilizumab therapy. **(a)** Annualized attack rates, with bars indicating mean estimates and whiskers indicating 95% CIs, **(b)** Expanded Disability Status Scale, with bars indicating median and whiskers indicating range **(c)** Modified Rankin Scale, with bars indicating median and whiskers indicating range, **(d)** Hauser and Ambulation Index (HAI), with bars indicating median and whiskers indicating range **(e)** EDSS score change over time **(f)** Distribution of mRS scores at different timepoint,(g) Distribution of HAI scores at different timepoint, **(h)** AQP4-IgG-titer at different timepoint, Each point represents an individual patient’s AQP4-IgG titer value. Gray lines connect serial measurements from the same patient, illustrating individual trajectories over time. Horizontal bars indicate median titers at each timepoint, with whiskers showing the full data range, **(i)** Median percentage of CD20+B cell at different timepoint in patients with prior B-cell depletion therapy, with whiskers indicating interquartile range, **(j)** Median percentage of CD20+B cell at different timepoint in patients without prior B-cell depletion therapy, with whiskers indicating interquartile range. For longitudinal comparisons, paired t-test/Wilcoxon test was used for two time points, and repeated-measures ANOVA/Friedman test was used for more than two time points, with *post-hoc* corrections as needed. ARR, Annualized attack rates, EDSS, Expanded Disability Status Scale, mRS, modified Rankin Scale, AQP4, aquaporin-4, SEM, standard error of the mean, ****p < 0.0001, ***p < 0.001,**p < 0.01,*p < 0.05 ns, not significant, p > 0.05.

Inebilizumab significantly reduced ARR from 1.496 (*95% CI*: 1.351–1.641) at baseline to 0.133 (*95% CI*: 0.062–0.205) at 12 months (p < 0.001) (**Figure 3A**).

The most recent treatments prior to inebilizumab initiation in the patient cohort were rituximab (RTX, n=24), mycophenolate mofetil (MMF, n=24), eculizumab (n=13), azathioprine (n=5), tacrolimus (n=4), ofatumumab (n=4), cyclophosphamide (n=1), intravenous immunoglobulin (n=1), ciclosporin (n=1), and tocilizumab (n=1). Among the 24 patients who had received RTX before switching to inebilizumab, reasons for switching included indication-driven change (54.2%, *13/24*), disease relapse (33.3%, *8/24*), and inadequate B-cell depletion (12.5%, *3/24*). In all 24 patients, adequate B-cell depletion (defined as <1% CD20+ B-cells) was achieved after switching to inebilizumab. However, 12.5% (*3/24*) still experienced further relapse. The mean ARR during RTX treatment was 0.452 (*95% CI*: 0.217–0.725), and it further reduced to 0.125 (0.125–0.250, p = 0.034) at 12 months on inebilizumab (online **Supplementary Figure 1A**). For the 24 patients previously on MMF, the reasons for switching to inebilizumab were disease relapse (70.8%, 17/24), indication-driven change (20.8%, 5/24), or adverse events (8.4%, 2/24), including pneumonia and elevated liver enzymes. Two patients experienced relapse during inebilizumab treatment. The mean ARR during MMF treatment was 0.816 (0.279-1.532), and it further reduced to 0.081 (0.00-0.177, p <0.001) at 12 months on inebilizumab (online **Supplementary Figure 1B**). Detailed data for the other treatment groups are provided in the online supplement.

### EDSS, mRS, and HAI

Patients starting inebilizumab during the remission phase showed a significant improvement only at 12 months (baseline: 2.5, range 0–7.5; 12 months: 2.0, range 0–4.0; p = 0.002), with no significant change at 6 months (2.5, range 0–6.0; p = 0.19) (**Figures 3B, E**). In contrast, the median EDSS scores of the total cohort decreased significantly from baseline (2.5, range 0.0–8.0) to 6 months (2.0, range 0–6.5; p = 0.02) and to 12 months (2.0, range 0–4.5; p < 0.001). A similar trend was observed in patients initiating inebilizumab during an acute attack phase (time from recent attack to treatment <3 months), with significant reductions from baseline (3.0, range 0–8.0) to 6 months (2.5, range 0–6.5; p = 0.04) and to 12 months (2.0, range 0–4.5; p < 0.001). The proportion of participants with minimal or no disability (mRS score <2) increased over time, with median scores remaining at 1 but showing significant range reductions at 12 months (0-4; p = 0.006) (**Figures 3C, F**). The HAI remained stable throughout, with median scores of 1.0 at baseline (range 1-11), 6 months (range 1-8), and 12 months (range 1-3) (**Figures 3D, G**).

### Safety

#### Persistence

During the 12-month follow-up, eleven patients discontinued inebilizumab due to pneumonia (*n* = 5), relapse (*n* = 5), or pregnancy (*n* = 1). The 12-month drug retention rate was 88.9% (95% CI: 82.8–95.5%) (**Figure 2B**). Subsequent therapies among these patients included satralizumab (*n* = 7), oral prednisolone 10 mg/day (*n* = 2), mycophenolate mofetil (*n* = 1),and rituximab (*n* = 1).

### Adverse events

AEs during follow-up are summarized in **Table 3**. At least one AE was reported in 113 of 136 patients (83.1%), with a mean incidence of 0.695 events per person-year (95% CI: 0.567–0.824). The most frequent AEs were infusion-related reactions (IRR, 64.7%, 88/136; mean incidence: 0.542 per person-year, 95% CI: 0.428–0.655) and infections (52.9%, 72/136; mean incidence: 0.443 per person-year, 95% CI: 0.341–0.545). Severe AEs occurred in 8.1% of patients (11/136; mean incidence: 0.068 per person-year, 95% CI: 0.028–0.108), including pneumonia (n=7), upper respiratory tract infection (n=2), hepatic function abnormality (n=1) and gastroenteritis (n=1).

**Table 3:** Adverse events in all participants during inebilizumab treatment.

Adverse events	Participants, n (%)	Mean (95% CI) incidence per person-year
At least one treatment-emergent adverse event	113 (83.1)	0.695 (0.567-0.824)
At least one adverse event graded as severe	11 (8.1)	0.068 (0.028-0.108)
Permanently discontinued due to treatment-related adverse event	5 (3.6)	0.031 (0.004-0.058)
Most frequent treatment-emergent adverse events^†^		
Infusion-related reaction	88 (64.7)	0.542 (0.428-0.655)
Upper respiratory tract infection	48 (35.2)	0.295 (0.212-0.379)
Urinary tract infection	17 (12.5)	0.105 (0.055-0.154)
Alopecia	15 (11.0)	0.092 (0.046-0.139)
Adverse events of special interest		
At least one adverse event of special interest	110 (80.8)	0.677 (0.550-0.803)
Infusion-related reaction	88 (64.7)	0.542 (0.428-0.655)
Infection	72(52.9)	0.443 (0.341-0.545)
Herpes zoster	7 (5.1)	0.043 (0.011-0.075)
Herpes simplex	6 (4.4)	0.037 (0.007-0.066)
Covid-19	4 (2.9)	0.025 (0.000-0.049)
Influenza	4 (2.9)	0.025 (0.000-0.049)
Bronchitis	3 (2.2)	0.018 (0.000-0.039)
Opportunistic infection	5 (3.6)	0.031 (0.004-0.058)
Fungal respiratory tract infection	3 (2.2)	0.018 (0.000-0.039)
Myalgia	7 (5.1)	0.043 (0.011-0.075)
Dyslipidemia	5 (3.6)	0.031 (0.004-0.058)
Leukopenia	4 (2.9)	0.025 (0.000-0.049)
Headache	4 (2.9)	0.025 (0.000-0.049)
Hepatic function abnormality	3 (2.2)	0.018 (0.000-0.039)
Back pain	3 (2.2)	0.018 (0.000-0.039)
Tumor	0	0
Death	0	0

All participants receiving ≥1 dose of inebilizumab were included in the safety analysis.

†Adverse events that occurred in >10% of participants

The most frequent IRRs were somnolence (52.9%, 72/136) and fatigue (35.3%, 48/136), potentially attributable to premedication with promethazine injection for allergy prophylaxis. Other IRRs—including pyrexia (10.3%, 14/136), myalgia (6.6%, 9/136), and chills (5.9%, 8/136)—occurred less frequently; all IRRs were grade 1.

The most common infection types were upper respiratory tract infection (35.2%, 48/136) and urinary tract infection (12.5%, 17/136). Eight patients developed pneumonia: three bacterial, two Pneumocystis, one Legionella, one yeast-related, and one COVID-19 requiring intubation and mechanical ventilation. Among these patients, one had previously received rituximab for 18 months, one had been treated with mycophenolate mofetil for one month, one had received azathioprine for 60 months, and the remaining five were treatment-naïve. Except for one bacterial case, all pneumonias were severe, requiring hospitalization and intravenous antibiotics; all patients recovered fully with treatment. Five patients developed opportunistic infections, including one case of Pneumocystis pneumonia with concurrent oral candidiasis, one case of Pneumocystis pneumonia alone, one case of Legionella pneumonia, one case of yeast pneumonia, and one case of fungal vaginitis.

Inebilizumab treatment induced a decline in lymphocyte counts from baseline (median 2.0) to Day 15 (1.7, p=0.022), Month 6 (1.5,p=0.004), and Month 12 (1.4, p<0.001), while median neutrophil counts remained stable until Day 15 (Baseline: 6.2 ×10^9^/L vs. Day 15: 6.3 ×10^9^/L, p=0.837) before significantly decreasing at Month 6 (3.8 ×10^9^/L, p<0.001) and Month 12 (3.5 ×10^9^/L, p<0.001) (online **Supplementary Figure 2**). At the 12-month follow-up, lymphocyte count decreases were reported in 13 patients (Grade 1: n=5; Grade 2: n=8). Neutrophil-related AEs occurred in seven participants (Grade 1 neutrophil count decreased: n=2; Grade 2 neutrophil count decreased: n=2; Grade 3 neutrophil count decreased: n=1; Grade 1 neutrophil count increased: n=1) (Online **Supplementary Table 2**).

**Figure 4:**
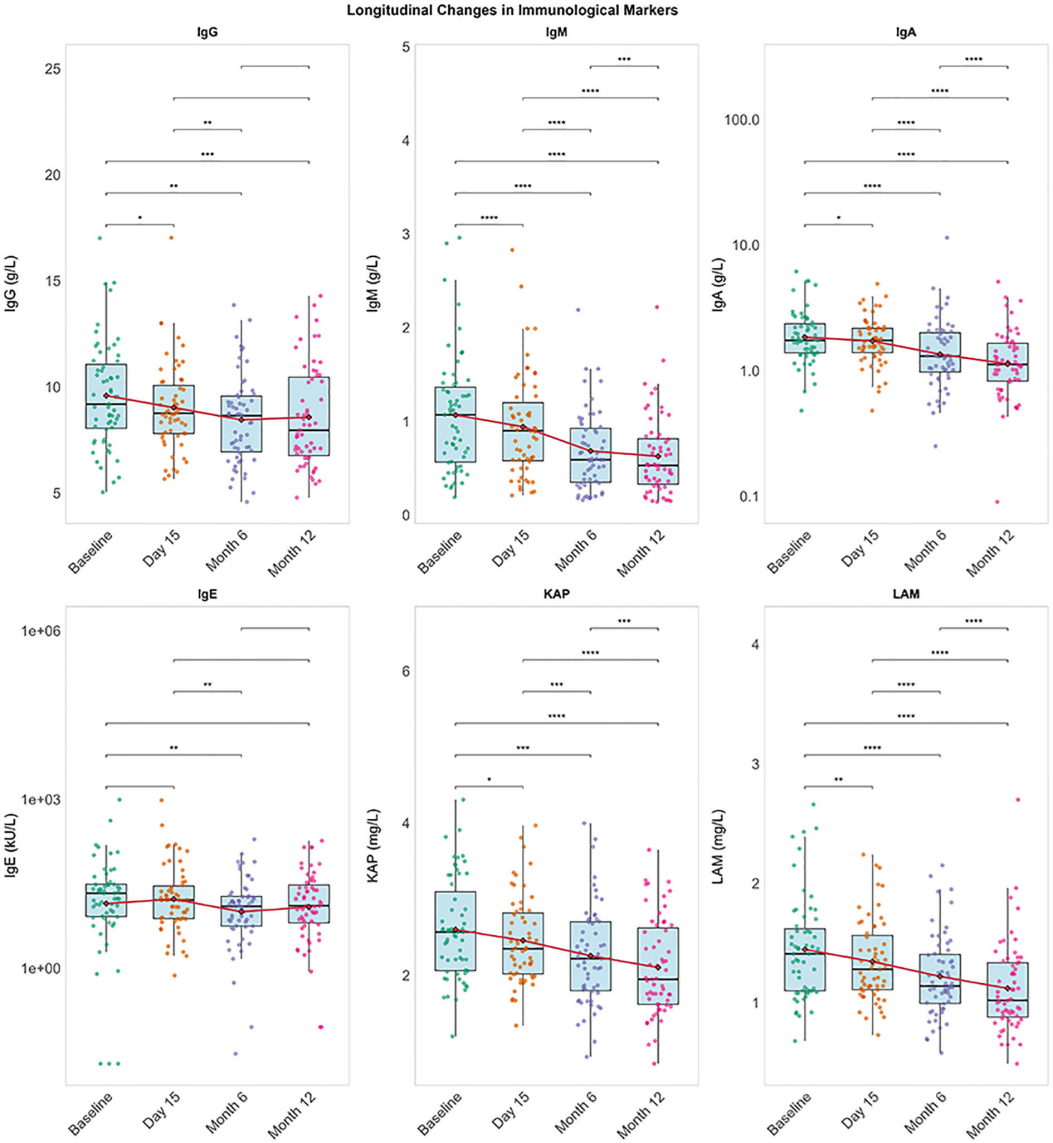
Longitudinal changes in serum immunological markers. Boxes: interquartile range, horizontal line: median, dots: individual values, red line & diamond: mean value, ****p < 0.0001, ***p < 0.001,**p < 0.01,*p < 0.05. Longitudinal within-group comparisons were conducted using the Friedman test, with *post-hoc* Dunn’s test correction for multiple comparisons.IgG, Immunoglobulin G; IgM, Immunoglobulin M; IgA, Immunoglobulin A; IgE, Immunoglobulin E; KAM, kappa free light chains; LAM, lambda light chain restriction.

Longitudinal analysis demonstrated significant reductions in blood immunoglobulin levels (IgG, IgM, IgA, IgE; all p ≤ 0.034 vs baseline) and free light chains (κ and λ; p < 0.001) over 12 months. The κ/λ ratio increased significantly from month 6 onward (month 12: p < 0.001 vs baseline), consistent with disproportionate suppression of λ chains (**Figure 4**). Serum IgG levels <5g/L were reported by five patients (three at baseline and two at Month 6), three patients received intravenous immunoglobulin supplementation.

No neoplasms, progressive multifocal leukoencephalopathy nor deaths occurred. Detailed AEs are available in the online **Supplementary Table 3**.

### Factors associated with severe pneumonia

Compared to patients without severe pneumonia, those who developed severe pneumonia during inebilizumab therapy had significantly higher BMI (median [range]: 25.0 [20–30] vs. 22.2 [15–38]; p = 0.046) and lower baseline IgG levels (6.09 [4.77–13.19] g/L vs. 9.56 [4.31–26.65] g/L; p = 0.011) (**Table 4**).

**Table 4:** Clinical characteristics and risk factors for severe pneumonia during inebilizumab treatment.

Clinical characteristics	Baseline characteristics comparison			Univariate analysis*		Multivariate analysis*	
	Without Severe Pneumonia N = 129^*^	Severe Pneumonia N = 7^*^	P-value	OR (95% CI)	P value	OR (95% CI)	P value
Age at treatment initiation, years, mean (SD)	39.0 (31.0–49.0)	44.0 (33.0–51.0)	0.531	1.02 (0.95, 1.08)	0.617	–	**-**
Age category, n(%)			0.232				
Age<55	113 (87.6)	5 (71.4)		Reference		Reference	
Age≥55	16 (12.4)	2 (28.6)		2.83 (0.38, 14.4)	0.237	3.09 (0.28, 27.7)	0.328
Gender, n(%)			0.100				
Female	120 (93.0)	5 (71.4)		Reference		Reference	
Male	9 (7.0)	2 (28.6)		5.33 (0.70, 29.0)	0.064	2.67 (0.21, 24.6)	0.422
BMI, kg/m², median (range)	22 (15–38)	25 (20–30)	**0.046**	1.19 (1.0, 1.44)	0.056	–	–
Weight classification, n(%)			**0.033**				
Normal	105 (81.4)	3 (42.9)		Reference		Reference	
Overweight/obesity	24 (18.6)	4 (57.1)		5.83 (1.21, 31.3)	**0.029**	2.87 (0.39, 20.5)	0.287
Baseline EDSS score	2.5 (0.0–8.0)	3.0 (0.0–8.0)	0.091	1.58 (1.07, 2.32)	**0.022**	-	**-**
EDSS category, n(%)			**0.031**				
EDSS <6.5	125 (96.9)	5 (71.4)		Reference		Reference	
EDSS ≥6.5	4 (3.1)	2 (28.6)		12.5 (1.49, 83.2)	**0.023**	25.4 (2.12, 334)	**0.013**
Baseline serum IgG level, median (range), g/L	9.6 (4.3–26.6)	6.1 (4.7–13.2)	**0.011**	0.57 (0.33, 0.86)	**0.005**	–	–
Baseline serum IgG category, n(%)			**0.009**				
IgG >6 g/L	122 (94.6)	4 (57.1)		Reference		Reference	
IgG ≤6 g/L	7 (5.4)	3 (42.9)		13.1 (2.23, 71.9)	**0.006**	14.2 (1.79, 136)	**0.007**
Serum IgG level decline rate, mean(SD), g/L*month†	0.45 (0.49)	0.34 (0.46)	0.584	0.59 (0.06, 3.20)	0.582	–	–
High-dose steroid pulse therapy, n(%)			0.716				
No	66 (51.2)	3 (42.9)		Reference	0.670	–	–
Yes	63 (48.8)	4 (57.1)		1.40 (0.30, 7.33)		–	–
Baseline oral steroid dosage, median (range), mg/day	30.0 (0.0–65.0)	35.0 (0.0–60.0)	0.410	1.02 (0.98, 1.06)	0.374	–	–
Prior immunosuppressant use, n(%)			0.459				
No	54 (41.9)	4 (57.1)		Reference		–	–
Yes	75 (58.1)	3 (42.9)		0.54 (0.10, 2.54)	0.432	–	–
Comorbidities, n(%)			1.000				
No	124 (96.1)	7 (100.0)		Reference	0.993	–	–
Yes	5 (3.9)	0 (0.0)		0.00 (0, inf)		–	–

*Data are presented as n (%), mean(sd) or median (range). Between-group comparisons were made using the χ² test, Fisher’s exact test, Student’s t-test, or the Mann–Whitney U test, as appropriate. Variables associated with the outcome (p < 0.20 in univariable analysis) were included in a multivariable logistic regression model. Backward stepwise selection (retention threshold: p < 0.20) was used for variable selection. Results are presented as Odds Ratios (OR) with 95% Confidence Intervals (CI).

†Calculated as the difference between the last follow-up and baseline values divided by the follow-up duration (in months).

BMI, body mass index; EDSS, Expanded Disability Status Scale; IgG ,Immunoglobulin G.Bold values indicate statistical significance (p < 0.05).

Univariable and multivariable analysis included age at treatment initiation (continuous and categorical), gender, BMI, baseline EDSS score (continuous and categorical), comorbidities (e.g., diabetes, cardiovascular diseases), baseline serum IgG level (continuous and categorical), serum IgG decline rate, prior high-dose steroid pulse therapy, baseline oral steroid dosage, and prior immunosuppressant use. Multicollinearity assessment revealed no significant concerns in the final multivariable logistic regression model. The VIF for all included variables ranged from 1.175 to 1.348, well below the conservative threshold of 5.0, indicating negligible multicollinearity among the predictors. It revealed that baseline EDSS ≥6.5 (OR 25.4, 95% CI: 2.12–334; p = 0.013) and IgG <6 g/L (OR 14.2, 95% CI: 1.79–136; p = 0.007) as independent risk factors for severe pneumonia (**Table 4**) during inebilizumab treatment.

### Comparison of outcomes among pretreatment subgroups

We analyzed the efficacy and safety of inebilizumab in treatment-naïve patients (n=58) and those switching from rituximab (RTX-switch, n=24) or mycophenolate mofetil (MMF-switch, n=24). Comparisons were limited to these three groups due to the small number of patients in other pretreatment subgroups.

The ARR was low and comparable across groups (p=0.712), as was the 12-month relapse rate (p=0.730). Notably, the RTX-switch group showed a greater median improvement in EDSS score (0.5; range: 0.0-3.0) than the treatment-naïve (0.0; range: 0.0-1.0) and MMF-switch groups (0.0; range: 0.0-0.5; p<0.001), despite higher baseline disability. Safety profiles were similar, with no significant differences in infection rates, though the RTX-switch group had a numerically lower overall infection rate (41.7%) than the treatment-naïve group (62.1%) (**Table 5**).

**Table 5:** Comparison of clinical outcomes among treatment-naïve, rituximaB-switch, and MMF-switch groups.

Clinical outcomes	Treatment-naïve (n=58)	RTX (n=24)	MMF (n=24)	P value
ARR during previous treatment, mean (95%CI)	1.154 (0.985-1.358)	0.452 (0.217–0.725)	0.816 (0.279-1.532)	**<0.001**
ARR during inebilizumab treatment, mean (95%CI)	0.069 (0.156-0.146)	0.125 (0.125–0.250)	0.081 (0.00-0.177)	0.712
ARR reduction, mean (95%CI)	1.146 (0.987-1.323)	0.191 (0.008-0.416)	0.561 (0.297-0.919)	**<0.001**
Relapse during inebilizumab treatment, n(%)	4 (6.9)	3 (12.5)	2 (8.3)	0.730
EDSS at inebilizumab initiation, median (range)	2.5 (1.0-4.0)	3.5 (1.0-7.0)	2.0 (1.0-3.0)	0.106
EDSS at last follow up, median (range)	2.0(0.0-4.0)	2.5 (0-3.5)	2.0 (0.0-3.0)	0.089
EDSS improvement, Median(range)	0.0 (0.0-1.0)	0.5(0.0-3.0)	0.0(0.0-0.5)	**<0.001**
At least one treatment-emergent adverse event, n(%)	52 (89.7)	18 (75.0)	17 (70.8)	0.089
At least one adverse event graded as severe	8 (13.8)	2 (8.3)	0 (0)	0.173
Infection rate during inebilizumab treatment, n(%)	36 (62.1)	10 (41.7)	12 (50)	0.230
Sever infection rate during inebilizumab treatment, n(%)	6 (10.3)	1(4.2)	1 (4.2)	0.692

*Data are presented as n (%), mean (sd) or median (range). Between-group comparisons across the three groups employed Chi-squared or Fisher’s exact tests for categorical variables, one-way ANOVA for normally distributed continuous variables, and the Kruskal-Wallis test for non-normally distributed variables.

†Calculated as the difference between the last follow-up and baseline values divided by the follow-up duration (in months).

ARR, annualized relapse rate; EDSS, Expanded Disability Status Scale.Bold values indicate statistical significance (p < 0.05).

### Serological biomarkers

AQP4-IgG titers were assessed every six months. The median baseline titer was 1:100 (range: negative to 1:1000). Titers remained largely stable following treatment (**Figure 3H**). Titer remain unchanged of 40.8% (38/93) at 6 months and 47.5% (29/61) at 1 year. At 6 months, 35.5% (33/93) of patients showed a reduction in AQP4-IgG titer, with 19 of them achieving seroconversion to negative, and 15.7% (3/19) become seropositive again at month 12. At the 1-year follow-up, the titer reduction rate was 29.8% (18/61), and 10 patients became seronegative. Titer increases were observed in 23.7% (22/93) and 22.9% (22/61) of patients at these respective time points.

No significant differences were observed between patients who ever became seronegative (n=22) and those who remained seropositive (n=71) in terms of ARR reduction (0.985 [95% CI 0.609–1.361] vs. 0.868 [0.643–1.092], p = 0.741) or EDSS improvement at month 6 (median 0.0 [range 0.0–1.5] vs. 0.0 [0.0–4.0], p = 0.150) and month 12 (median 0.0 [range 0.0–2.5] vs. 0.0 [0.0–4.0], p = 0.689). Seronegativity was not associated with relapse-free survival in our cohort (HR 0.72 [95% CI 0.16–3.33], p = 0.700) (Online **Supplementary Figure 3**).

The median baseline CD20+ B-cell percentage among lymphocytes was 0.88% (range 0-26.61) in patients with prior B-cell depletion therapy (rituximab, n=24; ofatumumab, n=2) versus 18.02% (3.07-37.92) in those without prior B-cell depletion. After the first inebilizumab infusion, CD20+ B-cells declined to 0% (0-2.20; p=0.023) and 0.09% (0-2.1; p<0.001) in these respective groups.

At month 6 (pre-third infusion), patients with prior B-cell depletion maintained 0% CD20+ B-cells (0-1.09), compared to 0.01% (0-12.72) in patients without prior depletion. By month 12 (pre-fourth infusion), these values were 0.01% (0-8.40) and 0.04% (0-21.27), respectively (**Figures 3I, J**).

Adequate B-cell depletion (CD20+ B-cell percentage <1%) was achieved in 91.1% (124/136) of patients by day 15 (prior to the second inebilizumab dose). This depletion was maintained in 84.0% (109/119) at month 6 (prior to the third dose) and in 85.5% (74/87) at month 12 (prior to the fourth dose).

### MRI

The mean cumulative number of contrast-enhancing or new T2 MRI lesions decreased significantly from 1.98 (95% CI: 1.10–2.86) at baseline to 0.137 (95% CI: 0.055- 0.247; p < 0.001) at 12 months.

## Discussion

The current study provides real-world evidence of inebilizumab therapy in Chinese patients with NMOSD. The main findings were (1): Inebilizumab demonstrates significant efficacy in reducing annualized relapse rates (ARR) and improving disability outcomes (EDSS, mRS) in Chinese AQP4+ NMOSD patients. (2) During the first 12 months following inebilizumab initiation, 14 attacks occurred in thirteen (9.6%) patients; independent risk factors for relapse during the first year of treatment included younger disease onset (age ≤20 years), longer disease duration (>5 years), and a higher number of attacks in the 2 years preceding therapy initiation. For patients with previous treatment failure and those who have undergone rapid steroid tapering, relapse should also be carefully monitored. (3) While generally well-tolerated, severe pneumonia necessitates vigilant monitoring, particularly in patients with baseline EDSS ≥6.5 or IgG <6 g/L, and those with high BMI.

The 12-month relapse-free rate was 88.1%, consistent with pivotal trial data (11, 12). This was further supported by a reduction in the annualized relapse rate (ARR), which dropped from 1.49 pre-treatment to 0.13 within the first year of therapy. These results reinforce inebilizumab’s effectiveness in suppressing disease activity in this real-world setting. Importantly, inebilizumab treatment led to a significant reduction in disability at 12 months, as evidenced by a lower median EDSS score and a higher proportion of patients achieving minimal disability (mRS <2). To distinguish the drug’s effect from natural recovery, we analyzed patients treated during stable phases (>3 months post-relapse) and still observed significant EDSS improvement (median: 2.5 to 2.0, p=0.002). This indicates that the functional benefits of inebilizumab extend beyond mere relapse prevention, which is consistent with the disability outcomes reported in its pivotal clinical trial (14). An important future direction will be to determine through long-term observation whether this disability improvement effect is sustained or increases over time. In line with findings from the eculizumab NMOSD trials (15), the stability observed in the HAI likely reflects the unique characteristics of ambulatory deficits in NMOSD, which may require extended observation or specialized rehabilitation strategies for improvement.

Among the 136 patients,13 patients experienced 14 relapses within the first treatment year. Independent predictors of relapse included younger age at disease onset (≤20 years), longer disease duration (>5 years), and a higher number of attacks in the 2 years preceding inebilizumab initiation. These findings are consistent with established evidence that patients with clustered attacks are at high risk of relapse (18–20). Furthermore, a recent study examining relapses during monoclonal antibody therapy targeting B-cells in NMOSD also identified longer disease duration and a higher number of pre-treatment attacks as significant risk factors for breakthrough relapse (21). While that study did not implicate age at onset, our analysis identified younger onset age (≤20 years) as an independent risk factor. This observation resonates with Kim et al., who demonstrated that younger onset age predicts a poor response to AZA or MMF therapy (19), and with Liu et al., who reported that older age at disease onset are associated with a lower relapse rate in NMOSD (22).

Prior treatment failure and rapid corticosteroid tapering have also been reported to increase the risk of NMOSD attacks (23, 24). In our study, while both factors were associated with relapse in univariate analysis, neither retained significance in the adjusted model. In the N-Momentum study and its extension (11, 12), relapses occurred most frequently within the first six months after treatment initiation, with relapse frequency declining as treatment duration increased. This pattern suggests that concomitant corticosteroid use, combined with a gradual tapering strategy during the initial six months of inebilizumab therapy, may help patients navigate this high-risk relapse period more smoothly. As recommended by the Chinese expert consensus: for patients receiving oral corticosteroids at the initiation of inebilizumab, corticosteroid therapy should be maintained to ensure an overlap of at least six months between corticosteroid and inebilizumab administration (25, 26). The optimal steroid tapering strategy during the inebilizumab therapy, as well as the optimal overlap time between steroids and inebilizumab, still requires further large-scale studies to provide clarity.

Notably, in all patients who had switched from rituximab to inebilizumab, adequate B-cell depletion (<1%) was achieved after switching, accompanied by a further reduction in ARR. This finding is consistent with previous studies (27, 28). However, relapses still occurred in 12.5% (3/24) of this subgroup after transitioning to inebilizumab, reinforcing that B-cell depletion alone may not fully eliminate disease activity in all individuals with NMOSD, potentially implicating plasma cell longevity or other T-cell–related immunological pathways (8, 29–32).

During inebilizumab therapy, AEs occurred frequently (83.1%), though severe AEs were uncommon (8.1%). IRRs and infections represented the most prevalent AEs. The IRR incidence in our cohort (64.7%) exceeded that reported in the open-label period of the N-MOmentum trial (13%) (12). The most prevalent IRRs observed in our cohort were somnolence and fatigue, effects may associate with promethazine premedication. Notably, the frequencies of pyrexia, myalgia, and chills - reactions more likely attributable to inebilizumab itself - closely paralleled those reported in the pivotal N-MOmentum trial. The infection profile was consistent with N-MOmentum open-label data (12), dominated by upper respiratory tract and urinary tract infections. Opportunistic infections were reported in five patients in our cohort and two patients in the N-MOmentum trial extension study (12). Fungal pneumonia was the most common opportunistic infection in our cohort (n=3) and was also observed in one patient in the N-MOmentum trial. This underscores the need for vigilant monitoring and proactive management for high-risk patients with opportunistic pulmonary infections.

In our cohort, the most notable AE is severe pneumonia, including two bacterial pneumonias, two Pneumocystis infections, one Legionella pneumonia, one yeast-related pneumonia, and one COVID-19 pneumonia requiring intubation and mechanical ventilation. While all patients achieved full recovery with appropriate treatment, five patients subsequently discontinued inebilizumab therapy. We identified baseline IgG <6 g/L and EDSS ≥6.5 as independent predictors for severe pulmonary infection. Although increased disability consistently correlates with infection risk during B-cell depletion therapy (33, 34), the relationship between treatment-induced hypogammaglobulinemia and infections remains controversial. While a study of 1,000 anti-CD20-treated patients identified IgG <5 g/L as a predictor of severe infections (33), long-term RTX data (14-year follow-up) and the N-MOmentum trial found no clear association between immunoglobulin levels and severe infection incidence (11, 12). This discrepancy likely arises from variations in baseline immune status, IgG metabolic kinetics, or genetic ancestry among different study populations. Furthermore, N-MOmentum assessed overall infections whereas we focused specifically on severe pneumonia. Notably, our baseline IgG reflects pre-treatment immune status, while N-MOmentum measured IgG during therapy when protective plasma cells remain active. This distinction aligns with our finding that the IgG decline rate did not significantly differ between patients with and without severe pneumonia. Further real-world validation of the baseline IgG level and its association with severe pneumonia risk is warranted.

Limitations of this study include its observational nature, the short observation duration, and the lack of a comparator group. The median observation period of 13.8 months, while sufficient to assess initial efficacy and short-term safety, is inherently limited for evaluating long-term immunological consequences and rare or delayed adverse events. Specifically, the trajectory of hypogammaglobulinemia, its potential association with cumulative infection risk, and the durability of B-cell reconstitution after treatment cessation require extended follow-up. The sample size, while substantial for a real-world NMOSD study, limits the power to detect smaller effects or rarer safety signals. Longer-term follow-up is essential to fully understand the durability of response, optimal steroid bridging strategy to avoid early recurrence, long-term safety implications, and the trajectory of subclinical biomarker change during inebilizumab treatment.

## Data Availability

The original contributions presented in the study are included in the article/**Supplementary Material**. Further inquiries can be directed to the corresponding authors.
