# Supplementary material for: Outcomes and predictors of relapse and severe pneumonia in Chinese patients with AQP4-IgG-positive neuromyelitis optica spectrum disorder receiving inebilizumab: a prospective cohort study
Source: Front Immunol. 2026 Jan 12;16:1718896. doi: 10.3389/fimmu.2025.1718896 (PMC12832490; doi:10.3389/fimmu.2025.1718896)
Supplement: Supplementary file 1 [file DataSheet1.pdf]

Online supplemental material

Procedures

Intravenous inebilizumab was administered at a fixed dose of 600 mg on days 1 and 15, followed by maintenance dosing every 6 months thereafter.Treatment was discontinued if participants withdrew consent or if the investigator identified an adverse event that precluded further dosing, including an elevated liver aminotransferase concentration, severe anaphylaxis, a hypersensitivity reaction, an infusion reaction, or neutropenia, or severe infection or if the participant became pregnant.

At baseline, demographic characteristics (age, sex, and self-identified racial background) were systematically recorded and disease-specific parameters were retrospectively analyzed, encompassing clinical trajectory(date of onset, initial symptom profile),relapse history (total number of attacks with detailed phenotypic characterization) and immunotherapy history (including start and stop dates and reason for change in treatment).

Baseline and longitudinal assessments followed a standardized protocol with scheduled visits at baseline (Day 1), Day 15, and 6-month intervals thereafter. Venous blood sampling at all timepoints included, routine hematology(Complete blood count with differential), serum biochemistry(liver enzyme, renal profile, AQP4-IgG(quantification via fixed-cell-based assay with indirect immunofluorescence) immunoglobulin isotyping (IgG, IgM, IgA, IgE) and B-cell [CD20+]). All laboratory analyses were conducted at a central laboratory of Huashan Hospital using standardized operating procedures. MRI and biomarker assessments were performed by blinded technicians following predefined quality control protocols. Clinical assessments including the Expanded Disability Status Scale (EDSS), Modified Rankin Scale (mRS), Hauser and Ambulation Index (HAI) were systematically administered at baseline and every 6 months during follow-up.

MRI was performed at baseline and annually thereafter, using a 3T scanner (Discovery MR750W, GE Medical System) with an eight-channel phased-array head coil. Gadolinium-enhanced magnetic resonance imaging (MRI) was performed upon detection of disease relapse. Adverse events (hereafter abbreviated as AEs) were

systematically classified using the Medical Dictionary for Regulatory Activities (MedDRA v27.1), with coding performed according to system organ class and preferred term nomenclature. AE severity was graded using a 5-tier classification system: grade 1 (mild), grade 2 (moderate), grade 3 (severe), grade 4 (life-threatening), and grade 5 (fatal). Serious adverse events (SAEs) were defined as meeting any of the following criteria: (1) grade 4-5 severity, (2) requiring hospitalization or prolongation of existing hospitalization, or (3) resulting in persistent or significant disability/incapacity. All AEs underwent protocol-mandated assessment at every study visit. Causal relationship to the investigational product was initially determined by tow investigators independently.

Sample Size Calculation for 12-Month Relapse-Free Survival

Design: Single-arm study evaluating time to first relapse at 12 months (Kaplan-Meier analysis).

Formula:

$$n=\frac{Z^2 \cdot S(12) \cdot (1-S(12))}{d^2}$$

S(12)=0.85 (expected 12-month relapse-free survival, results from the open-label period of the N-MOmentum trial)

Z=1.96 (95% CI, two-tailed)

d=0.08 (±8% margin of error)

Calculation:

$$n=\frac{1.96^2 \cdot 0.85 \cdot 0.15}{0.08^2} \approx 77$$

The sample size was further adjusted to account for an anticipated 10% attrition rate. Based on this adjustment, the final required sample size is approximately 85 participants. This ensures adequate power to estimate the 12-month relapse-free survival rate with an expected value of 85% and a 95% confidence interval width of ±8%, while maintaining study validity despite potential participant dropout.

ARR

No relapses occurred in patients switching from eculizumab (n=13; all for indication-driven change) or ofatumumab (n=4; all for indication-driven change). Relapses were observed in 1 of 5 patients switching from azathioprine (reasons: relapse [n=3], indication-driven change [n=1], AE [n=1]), 2 of 4 switching from tacrolimus (all for relapse), and the single patient switching from tocilizumab (for relapse). No relapses occurred in the single patients switching from cyclophosphamide, IVIG, or ciclosporin.

Table 1: Data Collection Schedule

**Assessment content**	**Time Point of Assessment Plan**			
Follow-up after inebilizumab initiation (days)	Screening and enrollment	Day15	Day195	Day365
Number of Visits	1	2	3	4
Informed Consent	√			
Inclusion/Exclusion Criteria	√			
Basic Information of Patient	√			
NMOSD Disease Information	√	√	√	√
Laboratory Tests				
Complete blood count, Lipid profile, Function of liver and kidney	√	√	√	√
Immunoglobulin	√	√	√	√
CD20+B-cell percentage in lymphocyte	√	√	√	√
AQP4 Antibody Titer	√		√	√
Examinations				
Standard Electrocardiogram (ECG)	√		√	√
Chest Computed Tomography (CT)	√		√	√
Magnetic Resonance Imaging (MRI)†	√			√
Neurological Functional Scores				
Expanded Disability Status Scale (EDSS)	√		√	√
Modified Rankin Scale (mRS)	√		√	√
Hauser Ambulation Index (HAI)	√		√	√
Adverse event	√	√	√	√

† MRI examinations will be performed according to the following schedule and sequences:

(1) Baseline: Non-contrast MRI of the brain and cervical/thoracic spinal cord.

(2) At Relapse: MRI of the brain and cervical/thoracic spinal cord including both non-contrast and gadolinium-enhanced T1-weighted sequences.

(3) Annual Follow-up (every 12 months): Non-contrast MRI of the brain and cervical/thoracic spinal cord.

Table 2 Maximum Toxicity Grade in Lymphocyte and Neutrophil Counts at different time point

	**Baseline**	**Day 15**	**Month 6**	**Month 12**
**Toxicity grade, n (%)**	**Participants (N=136)**	**Participants (N=136)**	**Participants (N=119)**	**Participants (N=87)**
Lymphocyte count				
Grade 0	134 (98.6)	107 (78.7)	100 (84.0)	74 (85.1)
Grade 1	2 (1.4)	17 (12.5)	12 (10.1)	5 (5.7)
Grade 2	0 (0)	9 (6.6)	5 (4.2)	8 (9.2)
Grade 3	0 (0)	3 (2.2)	2 (1.7)	0 (0)
Neutrophil count				
Grade 0	107 (78.6)	130 (95.7)	117 (98.3)	85 (94.3)
Grade 1	17 (12.5)	2 (1.4)	2 (1.7)	2 (2.3)
Grade 2	9 (6.6)	1 (0.7)	0 (0)	2 (2.3)
Grade 3	3 (2.2)	3 (2.2)	0 (0)	1 (1.1)

Table 3 Infusion-related reaction and rare adverse events during inebilizumab treatment.

	Participants,n (%)	Mean (95% CI) incidence per person-year
Infusion-related reaction	88 (64.7%)	0.542 (0.428-0.655)
Somnolence	72 (52.9%)	0.443 (0.341-0.545)
Fatigue	48 (35.3%)	0.295 (0.212-0.379)
Dry Mouth	25 (18.4%)	0.154 (0.094-0.214)
Pyrexia	14 (10.3%)	0.086 (0.041-0.131)
Myalgia	9 (6.6%)	0.055 (0.019-0.092)
Chills	8 (5.9%)	0.049 (0.015-0.083)
Diaphoresis	8 (5.9%)	0.049 (0.015-0.083)
Hypotension	4 (2.9%)	0.025 (0.000-0.049)
Throat Discomfort	3 (2.2%)	0.018 (0.000-0.039)
Respiratory Distress	3 (2.2%)	0.018 (0.000-0.039)
Other		
Pruritus	7 (5.1%)	0.043 (0.011-0.075)
Rash	6 (4.4%)	0.037 (0.007-0.066)
Blurred Vision	3 (2.2%)	0.018 (0.000-0.039)
Diarrhea	2 (1.5%)	0.012 (0.000-0.029)
Gastroenteritis	1 (0.7%)	0.006 (0.000-0.018)
Gastroesophageal reflux	1 (0.7%)	0.006 (0.000-0.018)
Vomiting	1 (0.7%)	0.006 (0.000-0.018)
Gastric ulcer	1 (0.7%)	0.006 (0.000-0.018)
Fungal vaginitis	1 (0.7%)	0.006 (0.000-0.018)
Dental caries	1 (0.7%)	0.006 (0.000-0.018)
Onychomycosis	1 (0.7%)	0.006 (0.000-0.018)
Elevated blood sugar	1 (0.7%)	0.006 (0.000-0.018)
Eye pain	1 (0.7%)	0.006 (0.000-0.018)
Arrhythmia	1 (0.7%)	0.006 (0.000-0.018)

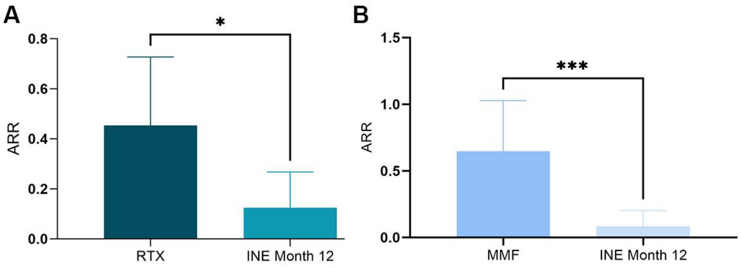

Figure1 Comparison of Annualized Relapse Rates Before and During Inebilizumab Treatment

Annualised attack rates, with bars indicating mean estimates and whiskers indicating 95%CIs.
Abbreviations: ARR , Annualized Relapse Rate; RTX, Rituximab; MMF, Mycophenolate mofetil; INE ,Inebilizumab.***p < 0.001,**p < 0.01,*p < 0.05, ns=not significant, p > 0.05

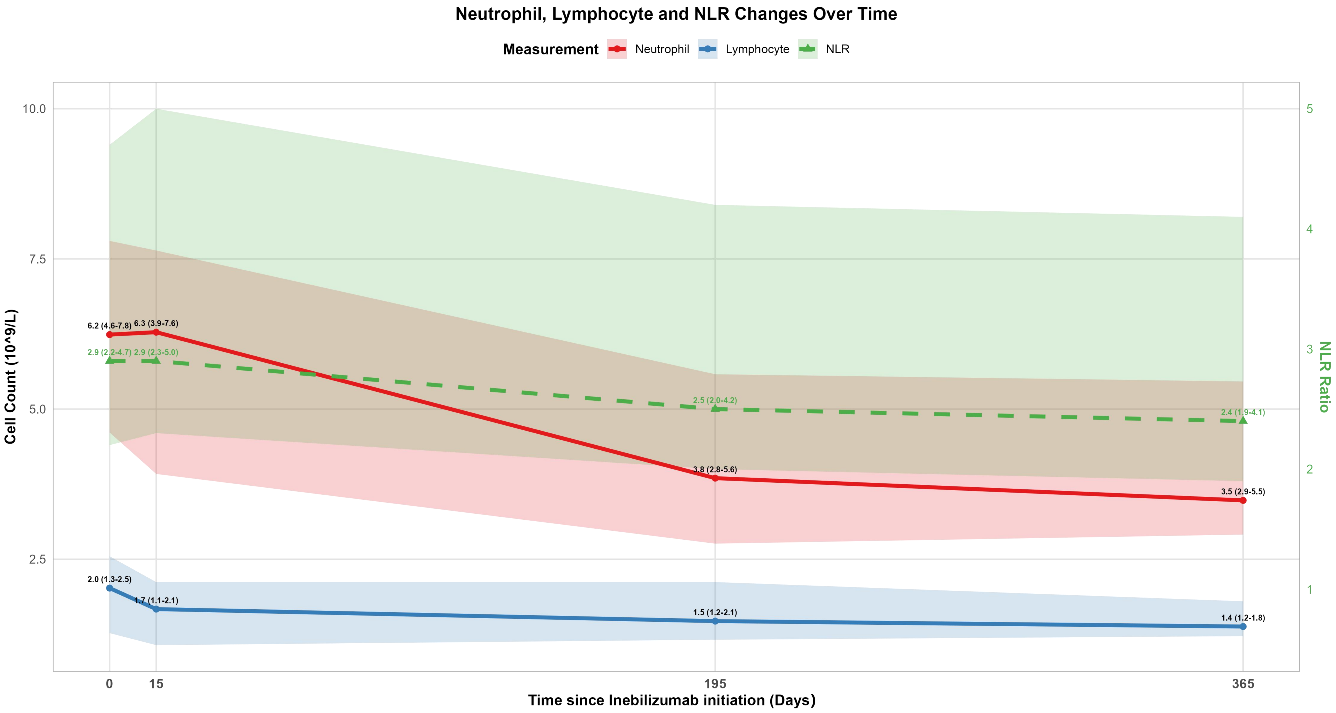

Figure 2. Longitudinal changes in neutrophil count, lymphocyte count, and neutrophil-to-lymphocyte ratio (NLR) following inebilizumab initiation.

Median values (symbols and connecting lines) with interquartile ranges (IQR, shaded bands) are shown for neutrophil counts (blue), lymphocyte counts (red), and NLR (green). The left y-axis represents cell counts ($x10^9$/L), while the right y-axis represents the NLR. Shaded bands denote the 25th to 75th percentiles. Data from 87 patients. Time points: Baseline, Day 15, Day 195, Day 365.

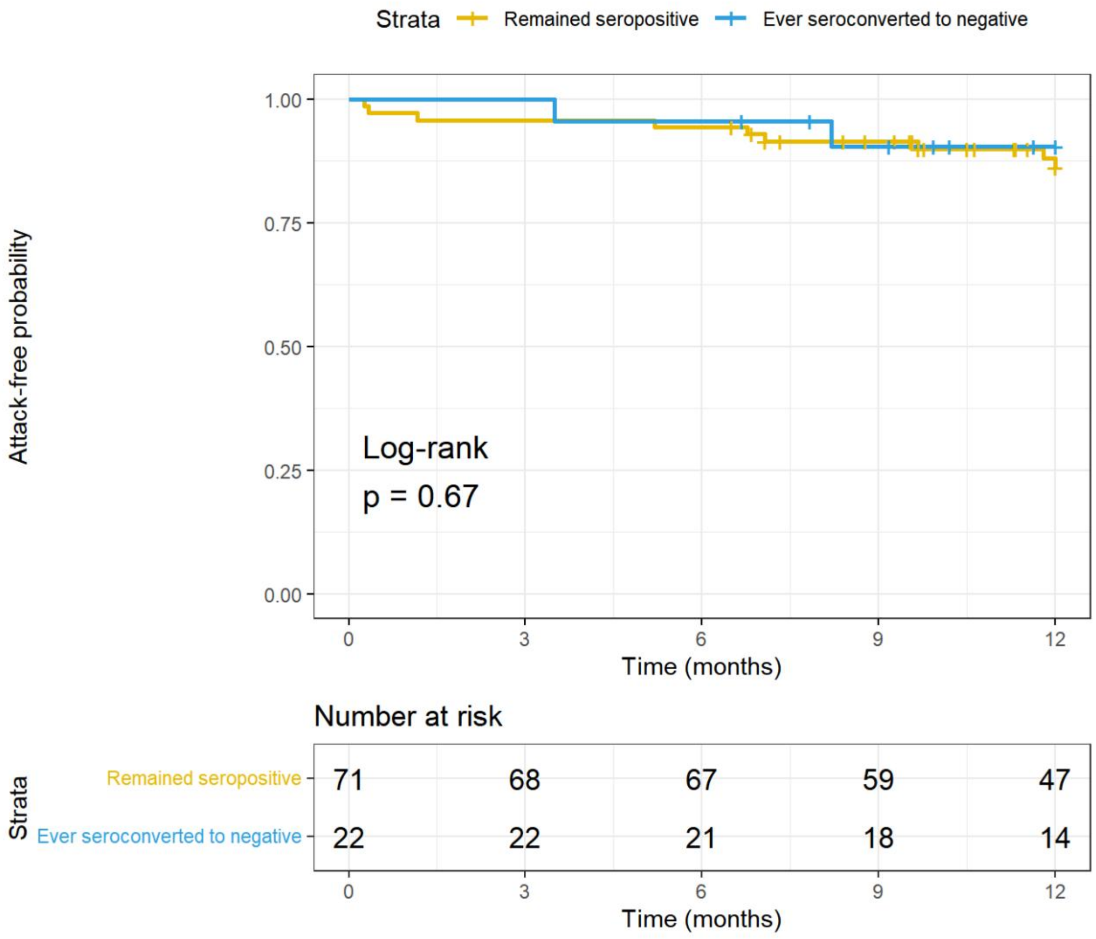

Figure 3 Kaplan–Meier curve estimating attack-free probability by serconversion status
